# Low Adherence to Mediterranean Diet Is Associated with Probable Sarcopenia in Community-Dwelling Older Adults: Results from the Longevity Check-Up (Lookup) 7+ Project

**DOI:** 10.3390/nu15041026

**Published:** 2023-02-18

**Authors:** Stefano Cacciatore, Riccardo Calvani, Emanuele Marzetti, Anna Picca, Hélio José Coelho-Júnior, Anna Maria Martone, Claudia Massaro, Matteo Tosato, Francesco Landi

**Affiliations:** 1 Department of Geriatrics and Orthopedics, Università Cattolica del Sacro Cuore, 00168 Rome, Italy; 2 Fondazione Policlinico Universitario “Agostino Gemelli” IRCCS, 00168 Rome, Italy; 3 Department of Medicine and Surgery, LUM University, 70100 Casamassima, Italy

**Keywords:** aging, muscle strength, nutrition, physical performance, handgrip strength, Medi-Lite, healthy diet, lifestyle, behavioral factors

## Abstract

Muscle strength is a relevant metric of aging. Greater adherence to Mediterranean diet is associated with better health outcomes across all life stages; however, evidence on the relationship between Mediterranean diet and muscle strength in older adults is inconclusive. In this study, we evaluated the relationship between adherence to Mediterranean diet and handgrip strength in a large sample of community-dwelling older adults from the Longevity Check-up 7+ project. A total of 2963 participants (mean age 72.8 ± 5.7 years; 54.4% women) were analyzed. Mediterranean diet adherence was evaluated using a modified Medi-Lite score and categorized as low (≤8), good (9 to 11), or high (≥12). Handgrip strength was categorized as normal or low according to cut-points by the European Working Group on Sarcopenia in Older People 2. Older adults with lower Mediterranean diet adherence had a significantly higher prevalence of probable sarcopenia (25.9%) than those with good (19.1%) or high (15.5%) adherence. The proportion of participants with probable sarcopenia increased with age, but it remained lower in the good and high adherence groups. Logistic regression showed that greater Mediterranean diet adherence was associated with a lower risk of probable sarcopenia. Older age, female sex, and physical inactivity were associated with a greater risk of probable sarcopenia. Our findings emphasize the positive association between healthy lifestyles, including adherence to Mediterranean diet, and physical function in old age.

## 1. Introduction

Muscle strength is a powerful metric of general health and a strong predictor of negative events across all life stages [1,2]. Muscle strength increases through adulthood, starts declining at middle age, and drops sharply at older age [3]. In older adults, muscle strength, mostly measured through handgrip strength testing, has been shown to predict major negative health-related events [4,5] such as disability, morbidity, and mortality [6,7]. Moreover, muscle strength assessment is the first step in the diagnostic algorithm for sarcopenia proposed by the European Working Group on Sarcopenia in Older People 2 (EWGSOP2) [8]. Handgrip strength correlates with muscle strength in other body districts and is, therefore, considered to be a reliable alternative to more complex strength tests (e.g., isometric peak torque of the knee extensors and flexors) [9]. Given its portability, affordability, safety, and ease of use, handgrip dynamometry is recommended by EWGSOP2 [8] and other expert groups [10,11] for the routine assessment of muscle strength. According to EWGSOP2, the detection of low muscle strength frames a condition termed “probable sarcopenia” [8]. The diagnosis of sarcopenia is confirmed when low muscle quantity or quality is also detected [8]. However, to avoid the under-recognition and undertreatment of sarcopenia, the prescription of therapeutic interventions (e.g., physical exercise, high-protein diet) should be considered in all cases of probable sarcopenia [8]. This implies that handgrip dynamometry is sufficient to identify and manage sarcopenia in everyday practice [8].

Several factors are associated with declining muscle strength in late life, including muscle-specific mechanisms [12], age-related diseases [13], and unhealthy lifestyle behaviors (e.g., physical inactivity) [14]. Nutrition plays a critical role in muscle physiology through the supply of energy substrates and molecules with anabolic properties [15,16]. Most research on the relationship between nutrition and muscle strength has focused on the intake of individual nutrients or dietary groups, particularly protein [15,17]. The influence of more complex dietary patterns on muscle strength parameters has been less investigated [18].

Mediterranean diet is a prototypical “healthy” diet [19,20]. In 1993, the Harvard School of Public Health, the Oldways Preservation Trust, and the World Health Organization developed the first Mediterranean diet pyramid, based on the food and lifestyle patterns of people living in Crete and Southern Italy in the 1960s. The typical Mediterranean diet is a balanced diet with moderate fat content (30–40%, mostly monounsaturated fats), 15–20% of proteins, and 50–60% of carbohydrates [21]. Grains, fruits, vegetables, and legumes are widely consumed; fish, poultry, milk and dairy products, and eggs are taken in moderation, while red meat and animal fats are consumed sparingly [21]. Olive oil is the primary source of added fat [21]. A greater Mediterranean diet adherence has been associated with several health benefits, including a reduced risk of cardiovascular disease [22], type 2 diabetes mellitus [23], cognitive decline [24], and cancer [25,26]. The mechanisms alleged to mediate the health benefits of Mediterranean diet are multifaceted and not yet completely understood. Studies suggest that specific components of the Mediterranean diet possess anti-inflammatory and antioxidant properties, stimulate cellular quality control processes, and attenuate mitochondrial dysfunction [27].

Evidence linking adherence to Mediterranean diet to muscle strength parameters in older adults is sparse [27,28,29]. In the present investigation, we therefore evaluated the association between various levels of adherence to Mediterranean diet and handgrip strength in a large cohort of old community dwellers enrolled in the Longevity Check-up (Lookup) 7+ project. We also explored whether greater adherence to Mediterranean diet would be independently associated with lower odds of probable sarcopenia.

## 2. Materials and Methods

The data analyzed in the present study were collected within the Lookup 7+ project. Lookup 7+ is an ongoing initiative coordinated by the Department of Geriatrics of the Università Cattolica del Sacro Cuore (Rome, Italy) and the Fondazione Policlinico Universitario “Agostino Gemelli” IRCCS (Rome, Italy). The study protocol and objectives are thoroughly described elsewhere [30,31]. Participants are considered eligible if they are 18 years or older. Exclusion criteria are self-reported pregnancy, refusal of capillary blood test, and unwillingness or incapacity to provide written informed consent. People visiting public spaces (e.g., exhibitions, shopping centers) or taking part in health promotion campaigns who agree to participate are administered a questionnaire and a short checkup to assess their adherence to healthy lifestyle habits. Modifiable cardiovascular factors and physical performance parameters are also assessed. Lookup 7+ activities have been held in small (100,000 residents), middle (100,000–250,000 residents), and large cities (>250,000 residents) to obtain a broad representation of individuals living in mainland Italy and major islands. Locations are chosen according to how many prospective volunteers may be recruited. In large cities, activities are held in several sites to achieve an adequate representation of the sociodemographic features of the population. Written informed consent is obtained from all participants before enrolment. The study protocol was approved by the Ethics Committee of the Università Cattolica del Sacro Cuore (protocol #A.1220/CE/2011) [30].

### 2.1. Anthropometry and Lifestyle Habits

Body weight and height were measured using an analog scale and a standard stadiometer, respectively. The body mass index (BMI) was then calculated by dividing body weight (kg) by the square of height (m^2^). Smoking status was categorized as (a) never smoked (never smoked or smoked fewer than 100 cigarettes in their lifetime), current smoker (smoked 100 cigarettes or more in their lifetime), and (c) former smoker (has smoked 100 cigarettes or more in their lifetime but quit at least 28 days prior to enrolment). For data analysis, smoking status was categorized as current or never/former smoker. Regular participation in physical activity or physical exercise was defined as engagement in activities at least twice a week for 30 min per session, over the previous year. Light walking, running, cycling, or swimming, as well as strength training with or without stretching exercises, were all taken into consideration. Participants who did not engage in any of those activities or did not meet the aforementioned requirements for frequency and/or duration were allocated in the physically inactive group.

### 2.2. Adherence to Mediterranean Diet

Nutritional data were collected using a food frequency questionnaire as part of the lifestyle questionnaire. To assess Mediterranean diet adherence, a modified version of the Medi-Lite score was calculated [32]. The Medi-Lite score was developed and validated by the Unit of Clinical Nutrition and Atherothrombotic Diseases of the “Careggi” Teaching Hospital (Florence, Italy) [32,33]. The tool was built using data collected in cohort studies that evaluated the association between adherence to a Mediterranean diet and health outcomes [33]. The Medi-Lite scoring system considers nine food categories: (1) fruit, (2) vegetables, (3) grains, (4) legumes, (5) fish and fish products, (6) meat and meat products, (7) dairy products, (8) alcohol, and (9) olive oil (Supplementary Table S1). For each food group traditionally included in the Mediterranean diet (i.e., fruits, vegetables, grains, legumes, and fish), a score ranging from 2 (greatest consumption) to 0 (lowest consumption) was attributed. For food items not included among typical Mediterranean diet ingredients, such as meat, meat derivatives, and dairy products, 2 points were assigned to the lowest consumption, 1 to intermediate consumption, and 0 to the greatest consumption. As for olive oil, 2 points were assigned to daily intake, 1 point to frequent use, and 0 for occasional consumption. Alcohol consumption was not recorded, and the corresponding item was therefore excluded from the Medi-Lite scoring. Consequently, the highest possible Medi-Lite score was 16 instead of 18. A score of 12 or above indicated high Mediterranean diet adherence, a score from 9 to 11 indicated good adherence, and a score of 8 or below indicated low adherence.

### 2.3. Muscle Strength Assessment

Muscle strength was assessed by handgrip strength testing using a handheld hydraulic dynamometer (North Coast Hydraulic Hand Dynamometer; North Coast Medical, Inc., Morgan Hill, CA, USA) following international guidelines, as previously described [3]. Handgrip strength testing was performed by trained investigators who had previously been certified by the standardization team of the “Sarcopenia and Physical fRailty IN older people: multi-componenT Treatment strategies” (SPRINTT) randomized clinical trial [34]. Inter-rater and test-retest reliability was evaluated at the time of investigator training. The results were consistent with the values reported in the literature. For the test, participants were asked to sit on a chair while keeping their shoulders relaxed in a neutral position and to flex their elbow at 90° close to their torso. The test was performed with the hand in a neutral position and thumbs up. Participants performed a familiarization trial prior to the actual test. Handgrip strength was measured in both hands and the highest reading (kg) was used for the analyses [35]. As mentioned earlier, in agreement with the EWGSOP2 consensus definition, low muscle strength values were considered to be indicative of probable sarcopenia [8]. Therefore, study participants were classified as having probable sarcopenia if their handgrip strength was <27 kg in men and <16 kg in women [8]. These cutoff points were chosen by EWGSOP2 as being two standard deviations (SDs) below the mean reference values of a young healthy population [36].

### 2.4. Statistical Analyses

Continuous variables were expressed as mean (SD), while categorical variables were reported as frequencies in absolute values and percentage of the total. Descriptive statistics were used to summarize the main personal, anthropometric, and functional characteristics of participants, according to Mediterranean diet adherence groups. Chi-squared tests and one-way analysis of variance, or Kruskal–Wallis H statistics when appropriate, were used to compare proportions and means of variables among participants with low, good, and high Mediterranean diet adherence. Logistic regression models were used to explore the relationship between adherence to Mediterranean diet and the prevalence of probable sarcopenia. To investigate whether Mediterranean diet adherence was associated with probable sarcopenia, we calculated the crude odds ratio (OR) with a 95% confidence interval (CI). To reduce confounding and improve the precision of estimates, logistic regression models were then built adjusting for age, sex, and other predictors previously associated with reduced muscle strength (e.g., BMI, physical activity). All analyses were carried out using the RStudio program, Version 4.2.2. (RStudio, Inc., Boston, MA, USA).

## 3. Results

Between 1 June 2015 and 30 December 2022, 4705 participants aged 65 years or older were enrolled across several Italian regions. Of them, 1742 were excluded for missing values in the variables of interest (147 for handgrip strength and 1638 for nutrition data). Therefore, a total sample of 2963 participants (54.4% women) was considered for the present investigation. The personal and anthropometric characteristics of the excluded participants did not significantly differ from those included in the analyses. The mean age of the study population was 72.8 years (SD 5.7) and 1612 (54.4%) were women. Adherence to Mediterranean diet was high in 812 (27.4%) participants, good in 1669 (56.3%), and low in 482 (16.3%). Table 1 shows participant characteristics according to Mediterranean diet adherence groups. Adherence to Mediterranean diet did not significantly differ between men and women. Participants with low adherence to Mediterranean diet were approximately one year older and showed a greater prevalence of unhealthy lifestyle factors, including smoking, overweight or obesity, and physical inactivity, compared with those with good and high adherence. Men had a mean handgrip strength of 34.9 (SD 8.3) kg, with no significant differences across Mediterranean diet adherence groups. Women had a mean handgrip strength of 20.9 (SD 5.2) kg, with significantly lower values in those with low Mediterranean diet adherence (*p* = 0.020). Five-hundred and sixty-nine (19.2%) participants were classified as having probable sarcopenia. Those with lower adherence to Mediterranean diet showed a higher prevalence of probable sarcopenia (25.9%) than participants with good (19.1%) and high adherence (15.5%) (*p* < 0.001).

**Figure 1:**
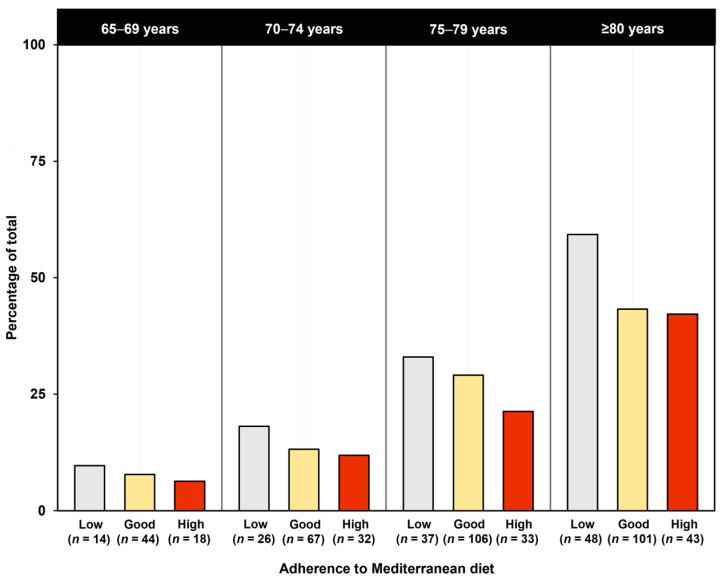
Proportions of participants with probable sarcopenia according to adherence to Mediterranean diet across age groups.

As shown in Figure 1, the prevalence of probable sarcopenia increased with age but remained consistently lower in the groups with good and high Mediterranean diet adherence relative to participants with low adherence.

In the unadjusted logistic regression model, both good (OR 0.68, 95% CI 0.54–0.86, *p* = 0.001) and high adherence (OR 0.53, 95% CI 0.40–0.70, *p* < 0.001) to Mediterranean diet were associated with a lower risk of having probable sarcopenia (Table 2). The association remained significant after adjusting for age and sex (OR 0.72, 95% CI 0.56–0.94, *p* = 0.021 for good Mediterranean diet adherence; OR 0.59, 95% CI 0.43–0.79, *p* = 0.002 for high Mediterranean diet adherence) (Table 2). After controlling for potential confounders, good (OR 0.71, 95% CI 0.55–0.92, *p* = 0.009) and high (OR 0.60, 95% CI 0.44–0.81, *p* < 0.001) adherence to Mediterranean diet were associated with a lower risk of probable sarcopenia. Predictors associated with a greater risk of probable sarcopenia were age and female sex in both the partially adjusted (age: OR 1.17, 95% CI 1.15–1.19, *p* < 0.001; female sex: OR 1.51, 95% CI 1.24–1.85, *p* < 0.001) and fully adjusted models (age: OR 1.17, 95% CI 1.15–1.19, *p* < 0.001; female sex: OR 1.50, 95% CI 1.22–1.84, *p* < 0.001) (Table 2). In the fully adjusted model, physical activity was associated with a reduced risk of probable sarcopenia (OR 0.69, 95% CI 0.56–0.85, *p* < 0.001). No significant associations with any other parameters were found.

## 4. Discussion

In the present study, we explored the association between adherence to Mediterranean diet and muscle strength in a large cohort of community-dwelling older adults using data from the Lookup 7+ project. We also assessed whether low adherence to Mediterranean diet was associated with a greater risk of probable sarcopenia. Our findings showed that female participants who reported low adherence to Mediterranean diet had lower handgrip strength values than those with good or high adherence. Older adults with low adherence to Mediterranean diet had a greater risk of probable sarcopenia after adjustment for potential confounders. Increasing age and female sex were also significant predictors of probable sarcopenia. Engagement in physical activity or exercise was instead associated with decreased odds of probable sarcopenia.

Our findings showed that most of the older adults enrolled in the study had high or good adherence to Mediterranean diet (83.7%). This observation is in keeping with findings of a recent systematic review that reported a higher prevalence of moderate adherence to a Mediterranean diet in older adults across European Mediterranean countries [37]. In a large cohort of 3131 individuals aged 65+ from the Mediterranean Islands (MEDIS) study, the mean level of adherence to Mediterranean diet was found to be moderate according to the MedDiet Score [38]. In older adults from the Italian Nutrition & Health Survey (INHES), in a 3-year telephone-based survey on nutrition and health with respondents from all over Italy, a moderate adherence to Mediterranean diet was observed [39]. In the INHES cohort, greater adherence to Mediterranean diet was found in middle-aged men (50–64 years) with a higher educational level who were living in Southern Italian regions. In a random sample of 5632 individuals aged 65–84 years from the Italian Longitudinal Study on Aging (ILSA), low adherence to Mediterranean diet was observed in 31.9% of the sample, while moderate and high adherence was reported by 26.1% and 41.7% of participants, respectively [40]. Interestingly, higher adherence to Mediterranean diet was associated with a lower risk of all-cause mortality. In a cross-sectional study involving participants from the Multicentrica Italiana Colelitiasi (MICOL) project assessed twice 20 years apart, the overall adherence to Mediterranean diet was found to be moderate [41]. However, rates of adherence decreased over time, particularly in younger participants [41]. A survey conducted within the Mediterranean Diet and Wellbeing (MeDiWeB) consortium and involving 3145 adults from seven Southern European countries (Bulgaria, Cyprus, Greece, Italy, Portugal, Republic of North Macedonia, and Spain) found that 68.3% of participants were classified as having moderate adherence to Mediterranean diet [42]. The adherence score, estimated using the 14-item Mediterranean Diet Adherence Screener (14-MEDAS), was found to slightly increase with aging and in women. Low adherence to Mediterranean diet (assessed with Medi-Lite) was documented in older adults from a nationwide cross-sectional study conducted in Malta island [43]. In a sample of non-selected Italians who completed the web version of the Medi-Lite score, moderate adherence to Mediterranean diet was reported across all age groups considered [44]. Finally, high adherence to Mediterranean diet was observed in older adults from the Mediterranean healthy Eating, Aging and Lifestyle (MEAL) study using the Medi-Lite score [45]. Collectively, our study confirms that a substantial level of adherence to Mediterranean diet is prevalent in Italian older adults, although the heterogeneity in the adherence indexes used hinders comparison across studies [37].

Emerging evidence supports the beneficial effects of the Mediterranean diet on musculoskeletal and functional parameters in older adults [27,28,29]. A recent meta-analysis by Coelho-Junior et al. [27]. showed that high adherence to Mediterranean diet was cross-sectionally associated with faster walking speed and greater knee muscle strength, besides better global cognition, in community-living older adults. However, conflicting results were reported on the relationship between adherence to Mediterranean diet and handgrip strength [27]. In a cohort of 84 community-dwelling older women from the PERsonalised ICT Supported Services for Independent Living and Active Ageing (PERSSILAA) project, 21.4% of participants reported low adherence to Mediterranean diet, which was associated with lower handgrip strength values [46]. Higher scores on an adapted Mediterranean diet assessment tool were related to a reduced probability of low handgrip strength in a cross-sectional study conducted as part of the Korea National Health and Nutrition Examination Survey (KNHANES) [47]. Over a 3-year period, higher Mediterranean diet scores were associated with better functional performance (i.e., faster walking speed and greater knee extension strength) and greater muscle mass in women aged 65–72 years from the Osteoporosis Risk Factor and Prevention-Fracture Prevention Study (OSTPRE-FPS) [48]. However, the association between adherence to Mediterranean diet and handgrip strength was not statistically significant. In a cohort of 182 German community-dwellers aged 75+ years, low Mediterranean diet adherence was associated with slow walking speed, physical inactivity, and physical frailty [49]. Although the “low grip strength” criterion of the frailty phenotype was the most prevalent among old, frail participants, its association with the Mediterranean diet score did not reach statistical significance. In a cohort of 690 community-living persons (≥65 years) enrolled in the Invecchiare in Chianti (InCHIANTI) study, a higher adherence to a Mediterranean-style diet was cross-sectionally associated with a lower probability of physical inactivity and better mobility, but not muscle strength [50]. Over six years of follow-up, participants with higher Mediterranean diet scores had a lower risk of incident physical frailty than did those with low adherence [50]. 

Collectively, our findings corroborate existing evidence on the positive effects of the Mediterranean diet on muscle function parameters in old age. Inconsistencies in the relationship between Mediterranean diet adherence and handgrip strength across studies may be attributed to the heterogeneity of study protocols (e.g., dietary adherence scoring, handgrip strength cutoffs, characteristics of study participants). 

Another result of the present study that deserves discussion is the significant association between engagement in physical activity or exercise and a lower risk of probable sarcopenia. This finding supports experts’ opinion that physical activity should be the first-line therapy to counteract age-related sarcopenia [51,52,53,54]. Numerous studies have reported that different exercise training modalities might improve upper- and lower-limb muscle strength in older adults [55,56,57,58,59,60,61]. Specific attention has been paid to resistance training-type exercise [62], since exercise aspects may be safely and easily adjusted to stimulate type II muscle fibers and produce significant improvements in muscle strength [62]. An increasing number of studies have also highlighted the potential beneficial effects of high-speed resistance training [51,63]. Pooled results of a meta-analysis that investigated the effects of resistance training on physical performance tests in frail older adults found improvements ranging from 6.6 to 37.0% in isometric and dynamic muscle strength measures, according to exercise and participant characteristics [64]. Other investigations reported no or marginal effects of exercise training on handgrip strength. For instance, Bernabei et al. [34] conducted a multicentric clinical trial that examined more than 1500 community-dwelling older adults of 70+ years across Europe. The trial involved a multicomponent intervention that included supervised, moderate-intensity physical activity twice weekly and personalized nutritional counseling. The results indicated that handgrip strength increased significantly in women after 24 months of intervention, but not in men. Taken as a whole, the present findings and those available in the literature suggest that combining a nutritional approach based on the Mediterranean diet with adequate exercise training might be an effective strategy to maintain or even improve handgrip strength.

**Figure 2:**
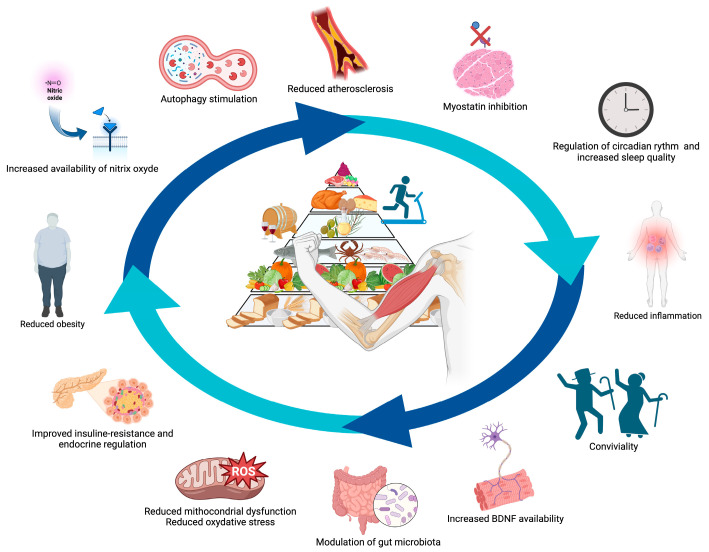
Possible mechanisms whereby the Mediterranean diet combined with physical activity benefits muscle health. Abbreviation: BDNF, brain-derived neurotrophic factor; ROS: reactive oxygen species. Created with BioRender.com, accessed on 30 January 2023.

Multiple components of the Mediterranean diet may contrast factors associated with age-related muscle strength decline, including malnutrition, oxidative stress, inflammation, endothelial dysfunction, and insulin resistance (Figure 2) [65,66,67,68,69].

The Mediterranean diet supplies a plethora of vitamins (e.g., β-carotene, vitamin C, vitamin E), minerals, and polyphenols, with antioxidant, anti-inflammatory, and pro-autophagic properties [67,70,71,72]. For instance, the PREvencion con DIeta MEDiterranea (PREDIMED) study found a decrease in inflammatory biomarkers, such as interleukin 6, C-reactive protein, and leukocyte adhesion molecules, in participants with higher Mediterranean diet adherence [73]. The Mediterranean diet also increases nitric oxide bioavailability by incorporating foods rich in both nitric oxide synthase (NOS) substrates (e.g., l-arginine, nitrates) and positive NOS modulators (e.g., vitamin C, polyphenols, omega-3 fatty acids) [74]. Noticeably, the Mediterranean diet lowers cholesterol and improves blood lipid profile [75]. People with a higher risk of cardiovascular disease tend to be frailer [76] and have more geriatric syndromes [77]. In addition, sarcopenia is positively associated with global atherosclerotic burden [78]. High-fat diets affect skeletal muscle through a variety of mechanisms. Lipid depots between and within muscle cells (myosteatosis) cause a “perfect storm” of mitochondrial dysfunction, increased oxidative stress, diminished insulin resistance, increased systemic inflammation, lipotoxicity, fiber atrophy, and damage to satellite cells [79]. Data also suggest that certain Mediterranean diet components, such as extra virgin olive oil, may increase the production of insulin-like growth factor 1 (IGF1) [80]. IGF1 is a hormone that regulates prenatal development, child growth, and muscle regeneration. Its availability declines with age or in conditions such as uncontrolled diabetes and obesity. A reduction in IGF1 blunts muscle protein synthesis and increases the production of myostatin, a negative regulator of muscle growth [81,82].

Despite reporting intriguing findings, the present study has limitations that should be acknowledged. First, the cross-sectional design precludes the possibility of drawing cause-and-effect implications between Mediterranean diet adherence and probable sarcopenia. Second, although handgrip strength was measured by trained investigators and following a standardized protocol, activities performed before its assessment (e.g., eating, carrying bags, walking) may have altered the test results. Third, no information on alcohol consumption was registered. Fourth, data collected did not include information on disease conditions, such as osteoarthritis or musculoskeletal and neurologic disorders, that may impact muscle strength. However, due to the study settings and the characteristics of the participants, it can reasonably be excluded that acute or severe illnesses were present at the time of evaluation. Fifth, the study population included virtually only Caucasians and the results may not be applicable to other racial groups. Sixth, the present investigation only involved older adults living in the community; thus, the results may not be generalizable to other settings. Seventh, several validated questionnaires exist to assess adherence to the Mediterranean diet, such as the MedDiet score, PREDIMED score, MSDPS (Mediterranean-style dietary pattern score), mMDS (modified Mediterranean diet score), and others. However, no tool proved to be superior to others. Eighth, the health benefits provided by the Mediterranean diet are thought to be conveyed by a combination of nutritional aspects and lifestyle and sociocultural elements, such as regular physical activity, adequate rest, and conviviality [83]. Whether the risk of probable sarcopenia is influenced by a Mediterranean diet as a lifestyle pattern or by dietary aspects alone warrants further study.

## 5. Conclusions

In the present investigation, we explored the association between Mediterranean diet adherence and muscle strength in a large sample of community-dwelling older adults. Our findings indicate that lower Mediterranean diet adherence scores were associated with lower handgrip strength and a higher risk of probable sarcopenia. Future studies are needed to establish whether lifestyle interventions including Mediterranean diet improve muscle strength in old age.

## Figures and Tables

**Table 1:** Characteristics of study participants according to adherence to Mediterranean diet.

	Low Adherence (n = 482)	Good Adherence (n = 1669)	High Adherence (n = 812)	Total Sample (n = 2963)	*p*
Age, years	73.5 (5.9)	72.8 (5.8)	72.5 (5.5)	72.8 (5.7)	0.034
Sex, female	275 (57.1%)	887 (53.1%)	450 (55.4%)	1612 (54.4%)	0.429
Smokers	81 (16.7%)	245 (14.6%)	91 (11.2%)	417 (14.0%)	0.030
BMI, kg/m^2^	26.6 (4.0)	26.3 (4.0)	25.6 (3.8)	26.2 (4.0)	<0.001
Underweight (BMI < 18.5 kg/m^2^)	0 (0.0%)	7 (0.4%)	5 (0.6%)	12 (0.4%)	0.462
Overweight or obese (BMI ≥ 25 kg/m^2^)	299 (62.0%)	998 (59.8%)	430 (53.0%)	1727 (58.3%)	<0.001
Physically active	208 (43.2%)	840 (50.3%)	427 (52.6%)	1475 (49.8%)	0.012
Handgrip strength, kg					
Women	20.2 (5.2)	20.8 (5.1)	21.4 (5.3)	20.9 (5.2)	0.020
Men	33.5 (8.3)	35.2 (8.4)	35.1 (7.7)	34.9 (8.3)	0.307
Probable sarcopenia	125 (25.9%)	318 (19.1%)	126 (15.5%)	569 (19.2%)	<0.001

Data are reported as absolute numbers (percentages) for categorical variables, such as sex, smoking status, physical activity, BMI categories, and probable sarcopenia. Continuous variables are reported as means (standard deviations). Physically active: engagement in physical activity or exercise at least twice weekly for a minimum of 30 min per session. Probable sarcopenia: handgrip strength <27 kg in men and <16 kg in women. Abbreviation: BMI, body mass index.

**Table 2:** Unadjusted and adjusted logistic regression models exploring the association between adherence to Mediterranean diet and probable sarcopenia.

Characteristics	Unadjusted OR (95% CI)	*p*	Age- and Sex-Adjusted OR (95% CI)	*p*	Fully Adjusted OR (95% CI)	*p*
Adherence to Mediterranean diet						
Low	–	–	–	–	–	
Good	0.68 (0.54−0.86)	0.001	0.72 (0.56–0.94)	0.021	0.71 (0.55–0.92)	0.009
High	0.53 (0.40−0.70)	<0.001	0.59 (0.43–0.79)	0.002	0.60 (0.44–0.81)	<0.001
Age			1.17 (1.15–1.19)	<0.001	1.17 (1.15–1.19)	<0.001
Sex, women			1.51 (1.24–1.85)	<0.001	1.50 (1.22–1.84)	<0.001
Normal BMI (<25 kg/m^2^)					0.85 (0.69–1.05)	0.120
Smokers					0.89 (0.65–1.20)	0.500
Physically active					0.69 (0.56–0.85)	<0.001

Abbreviation: BMI, body mass index; CI, confidence interval; OR, odds ratio.

## Data Availability

The data that support the findings of this study are available on request from the corresponding author. The data are not publicly available due to their containing information that could compromise the privacy of the research participants.

## Supplementary Materials

The following supporting information can be downloaded at: https://www.mdpi.com/article/10.3390/nu15041026/s1, Supplementary Table S1: Medi-Lite Scoring System Used in the Study (Modified from [33]).
