# Supplementary material for: Low Adherence to Mediterranean Diet Is Associated with Probable Sarcopenia in Community-Dwelling Older Adults: Results from the Longevity Check-Up (Lookup) 7+ Project
Source: Nutrients. 2023 Feb 18;15(4):1026. doi: 10.3390/nu15041026 (PMC9959184; doi:10.3390/nu15041026)
Supplement: Supplementary file 1 [file nutrients-15-01026-s001.zip › nutrients-2222593-supplementary.pdf]

Supplementary Table S1 Medi-Lite Scoring System Used in the Study (Modified from [33]).

Food categories	Scores		
	**0**	**1**	**2**
Fruit	<1 portion/day	1–1.5 portions/day	>1.5 portions/day
Vegetables	<1 portion/day	1–2.5 portions/day	>2.5 portions/day
Legumes	<1 portion/day	1–2 portions/day	>2 portions/day
Cereals	<1 portion/day	1–1.5 portions/day	>1.5 portions/day
Fish and fish products	<1 portion/week	1–2.5 portions/week	>2.5 portions/week
Meat and meat product	>1.5 portions/day	1–1.5 portions/day	<1 portion/day
Dairy products	>1.5 portions/day	1–1.5 portions/day	<1 portion/day
Olive oil	Occasional use	Frequent use	Regular use
